# Narrow Band Solid-Liquid Composite Arrangements: Alternative Solutions for Phononic Crystal-Based Liquid Sensors

**DOI:** 10.3390/s19173743

**Published:** 2019-08-29

**Authors:** Nikolay Mukhin, Mykhailo Kutia, Aleksandr Oseev, Ulrike Steinmann, Stefan Palis, Ralf Lucklum

**Affiliations:** 1 Institute for Micro and Sensor Systems, Otto-von-Guericke-University Magdeburg, 39106 Magdeburg, Germany; 2 Department of Photonics, Saint Petersburg Electrotechnical University “LETI”, Saint Petersburg 197376, Russia; 3 Institute for Automation Engineering, Otto-von-Guericke-University Magdeburg, 39106 Magdeburg, Germany; 4 FEMTO-ST Institute, CNRS UMR-6174, Université de Bourgogne Franche-Comté, 15B, Av des Montboucons, 25030 Besançon, France

**Keywords:** phononic crystal sensor, liquid sensor, periodic composite structure, solid-liquid interaction, acoustic band structure, acoustic transmission spectra, detection of fluid properties, speed of sound

## Abstract

Periodic elastic composite structures attract great attention. They offer the ability to design artificial properties to advance the control over the propagation of elastic/acoustic waves. In previous work, we drew attention to composite periodic structures comprising liquids. It was shown that the transmission spectrum of the structure, specifically a well-isolated peak, follows the material properties of liquid constituent in a distinct manner. This idea was realized in several liquid sensor concepts that launched the field of phononic crystal liquid sensors. In this work we introduce a novel concept—narrow band solid-liquid composite arrangements. We demonstrate two different concepts to design narrow band structures, and show the results of theoretical studies and results of experimental investigations that confirm the theoretical predictions. This work extends prior studies in the field of phononic crystal liquid sensors with novel concepts and results that have a high potential in a field of volumetric liquid properties evaluation.

## 1. Introduction

Phononic crystals are nowadays broadly used to control, direct, and manipulate sound waves. Acoustic properties of such structures are defined not only by material properties of the structure constituents (at least two materials with different properties), but also by design (geometry, symmetry, periodicity) [1,2,3,4,5,6]. Propagation of elastic waves through phononic crystals features wavelength regions, within which sound cannot propagate through the structure, so-called bandgaps [7,8,9,10,11,12]. Defects in this regular structure can be designed in a way that they cause isolated defect modes inside the band gap [13,14,15,16]. Since the acoustic properties of these composite arrangements depend on material properties of structure constituents, variation of one material property causes a change in the transmission behavior of the whole structure [17,18,19]. Analyzes of phononic crystals constituting a liquid have been published in [20,21,22,23]. They indicate that a variation of properties of the liquid constituent changes their response in a distinct manner. These findings enable the field of phononic crystal liquid sensors that is merging the advantages of ultrasonic and microacoustic liquid sensor approaches [24].

First work was dedicated to phononic crystal cavity mode type of structures that were applying the concept of a Fabry-Perot type resonator with the liquid present in the resonant volume [24,25,26,27,28,29,30,31]. 1D and 2D bandgap structures create artificial bandgap type boundary conditions that support a high quality factor acoustic pressure resonance in a liquid-filled cavity. The resonating cavity works as a structure defect that disrupts the periodicity and opens a narrow transmission window that correspond to a cavity mode resonance. Liquid pressure resonances of regularly distributed resonators support the transmission through the structure in a narrow window corresponding to pressure resonance modes in cylindrical liquid filled volumes. The use of the acoustic methods is also attractive since the analyte is not a part of an electrical circuit (acoustically decoupled) that e.g., prevents the occurrence of a spark when measuring flammable liquids. 

A completely different concept of phononic crystal liquid sensor [32,33] was inspired by the extraordinary optical transmission in photonics. A regularly perforated solid plate immersed in a liquid exhibits extraordinary transmission of acoustic pressure wave at normal incidence at a certain frequency [34,35,36]. We have shown that this frequency changes with variation of sound velocity of the liquid. Our findings have revealed a controversial explanation of extraordinary transmission. Initial attempts were focused on finding the similarity of the effect with known background from photonics. On the other hand, Zubtsov and Lucklum have proven structural vibration behind extraordinary acoustic transmission, that was demonstrated as a collective work of resonances in each of the cylindrical inclusions and the resonance mode of the solid periodic arrangement. 

The phononic crystal liquid sensor concept in a microacoustic sensor has been realized by integration with surface acoustic wave (SAW) devices [37,38,39]. The phononic structure was implemented in a straightforward way by a periodic structure on the surface in direction of wave propagation. Further studies [40] have applied a periodic structure at the interface of a SAW device. Here the surface wave couples to structure resonance modes. This effect causes dips in the transmission spectrum. They correspond to the properties of the liquid inclusions. Planar fabrication technology introduces some constrains that results in a rather complex fabrication to reach the required performance [41].

Due to the similarity in both physics and design, so-called phoxonic crystals, i.e., a combination of photonic and phononic crystals in one structure, have also been realized as sensors [42,43].

The idea of phononic crystal liquid sensors with a tubular liquid-filled main component considers the prevailing geometry of pipes or vessels [44,45]. The concept that implements elastic waves in axial symmetry is rather promising and currently the part of ongoing research. 

Very recently we have introduced a different concept of the well-known quartz crystal microbalance (QCM) [46]. The QCM is coupled to a single or a set of liquid-filled cavity resonators. A phononic structure, i.e. a periodic arrangement of holes in a solid interfacial layer, is the key to keep the extremely high Q-factors of the QCM shear thickness mode and the pressure resonance mode of the liquid cavity. The readout of both resonances happens within a pre-selected window of the electrical admittance spectrum of the QCM.

The sensitivity of the speed of sound measurement with phononic crystal sensors can outperform well-established ultrasonic principles. However, sound velocity is typically not the final value of interest. Since it is linked to Gibbs Free Energy [47,48] ultrasonic velocimetry allows for detecting molecular interactions [49], for example in enzyme catalysis [50], or microstructural transitions [51]. 

In this work we extend the field of PnC liquid sensors with a concept of a phononic crystal liquid sensor. It is based on narrowband composite solid-liquid structures. In contrast to previous work, the concept does not rely on cavity type resonance. The composite periodic structure has narrow transmission/reflection bands governed by the constituting liquid. We demonstrate two different ways to obtain narrowband structures with composite solid-liquid periodic arrangements. We discuss possible ways to obtain an optimal design in terms of both frequency shift and bandwidth of the employed liquid resonances. The paper reports theoretical investigations and experimental verification of the proposed narrowband phononic crystal sensor concept.

## 2. Materials and Methods

### 2.1. Theoretical Aspects 

**Figure 1:**
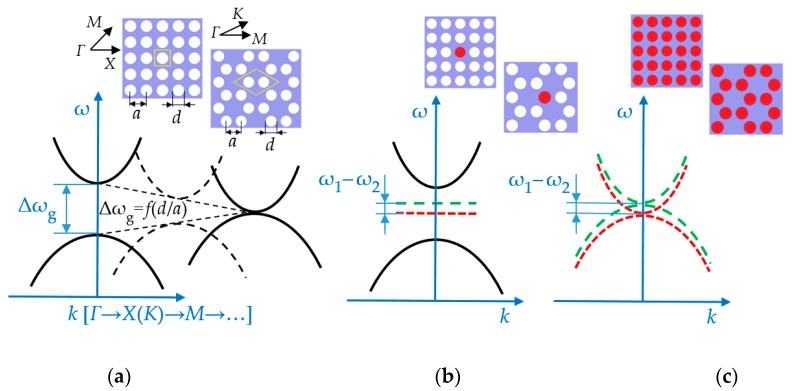
Basic approaches to design of phononic crystal based liquid sensor: (**a**) Schematic representation of the idea of a geometry defined bandgap width of phononic crystals based on two-dimensional arrangements of cubic or honeycomb symmetries; (**b**) an isolated liquid-filled hole point defect in a regular phononic crystal lattice; the liquid defect induces a band inside of broad bandgap phononic crystal; (**c**) periodic structure completely filled in with a liquid; here a narrow bandgap with a minimum or zero density of vibration states in the passband of overall structure is employed. Green and red (dotted lines) represent two different sound velocities of the liquid.

In this paper, we consider two contrary approaches to the design a narrowband phononic structure aiming at the detection of properties of the constituting liquid. Figure 1 schematically shows the band diagrams of two phononic crystals—the dependence of the angular frequency (ω) of the structure eigenmodes on the wave vector (**k**) close to high symmetry points (for example, *Γ*, *K* or *X*, *M*). It is physically customary to refer to 2D symmetry points with a bar on top to distinguish them from the 3D case, but in this article we considered only the 2D case, therefore, the bar is omitted for the simplification. By selecting the symmetry of the phononic crystal and the aspect ratio (the diameter of the holes (*d*) to the lattice constant (*a*)), we can manipulate the width of bandgaps, as shown in Figure 1a. In this paper, we are interested in two opposite cases—the case of a narrow transmission band made in a wide bandgap arrangement and the case of a narrow bandgap in a broad passband of the structure. In the case of a phononic crystal with a wide bandgap (Figure 1b), we can create localized states with a liquid contributing structure eigenmodes, where the solid matrix itself does not support any vibrational modes. It can be realized by introducing a defect into the regular structure of a phononic crystal, here made as liquid-filled inclusion. It locally disrupts the structure periodicity and opens a narrow transmission band. The frequency of the defect mode is isolated from the modes of the solid matrix. It is determined by the diameter of the hole and the speed of sound of liquid. The frequency of the liquid resonance, therefore, follows the variation of the speed of sound of the liquid inclusion. In this contribution we focus on the design of a narrow band that is created by liquid inclusions and initiates composite structure modes. 

The second case is illustrated in Figure 1c. We create a very narrow bandgap (in the limit of zero width) with degenerative structure modes and hybridized modes. The terminology originally comes from solid state physics and describes the state of a system at which the disrupted state is more energy efficient than a coupled one. In this case, the structure exhibits a low density of acoustic states for the wavelengths that correspond to a narrowband.

The ω_1_ and ω_2_ on Figure 1b,c depend primarily on the mode number, the speed of sound in the liquid, and the hole diameter. In addition, their values can be shifted due to the interaction with the solid-state matrix. Therefore, in the general case, ω_1_–ω_2_ are not the same for different sensor designs.

The computations with numerical methods were carried out to develop the structure design with optimal sensor performance.

The propagation of acoustic waves in an elastic medium is determined by the equation:

(1) $$\rho_{S}\frac{\partial^{2}u_{i}\left(r,t\right)}{\partial t^{2}}=\sum_{j,m,n}\frac{d}{dx_{i}}\left[C_{ijmn}\frac{\partial^{2}u_{n}\left(r,t\right)}{\partial x_{m}}\right],$$

where $u_{i}$ are the components of the elastic displacement field; $C_{ijmn}$ is the elasticity tensor; $\rho_{S}$ is the density; **r** = (*x*, *y*, *z*) is the coordinate vector; *t* is time.

Given that the composite is a periodic structure, the Bloch theorem was used to determine eigensolutions. According to this theorem, the displacement vector can be represented as a product of the periodic function $u_{k}$ of the phononic crystal and the propagating wave with **k** being the wave vector:

(2) $$u\left(r,k\right)=u_{k}\left(r\right)\exp\left(-ikr\right),$$

The band diagram of regular periodic structures will be modified by liquid pressure resonances. Resonance modes can be found by solving the eigenmode problem for acoustic modes in a cylindrical hole. The Helmholtz equation is the basic equation for the pressure wave with harmonic solutions:

(3) $$\nabla \left(-\;\frac{1}{\rho_{L}}\nabla p\right)-\frac{\omega^{2}p}{\rho_{L}V_{L}{}^{2}}=0,$$

where $\rho_{L}$ is the fluid density; ω is angular frequency; $V_{L}$ is speed of sound in a liquid; *p* is pressure. To calculate viscosity losses, we use the Navier–Stokes equation [52,53] instead of the Helmholtz Equation (3).

Conditions at the boundaries of the “solid – liquid” section are as follows:

(4) $$F=-n_{s}p,$$

where $n_{s}$ is the normal vector directed from a solid; **F** is the force per unit area representing the load on the cylinder walls. At the same time, the equality of the normal components of the acceleration vector at the interface between two solid-liquid media must be maintained:

(5) $$\left(n_{f}\cdot u\right)\omega^{2}=-n_{f}\left(-\;\frac{1}{\rho_{L}}\nabla p+q\right),$$

where $n_{f}$ is the normal vector directed from the fluid volume; **u** is the mechanical displacement vector in a solid; **q** is the acceleration vector reported by the fluid.

A computation of eigenmodes and transmission spectra of periodic composite structures based on Equations (1)–(5) was carried out numerically with COMSOL Multiphysics (Comsol Multiphysics GmbH, 37073 Göttingen, Germany) software.

The band diagrams were computed by solving the eigenfrequencies problem for the periodic composite structure. To determine the transmission response, frequency computational domain simulations were carried out for a finite structure with longitudinal harmonic excitation at one boundary.

The complete structure was realized as a periodic system of hollow cylinders, a commonly accepted approach to build 2D phononic structures. It is also compatible with standard computational methods. The solid matrix material should preferably have high values of sound velocity and density, i.e., high acoustic contrast to the liquid. Additionally, the matrix material should have low mechanical losses and must be chemically inert to the liquids studied. Following this requirements, stainless steel was found to be an optimal solution for this study and foreseen applications.

**Figure 2:**
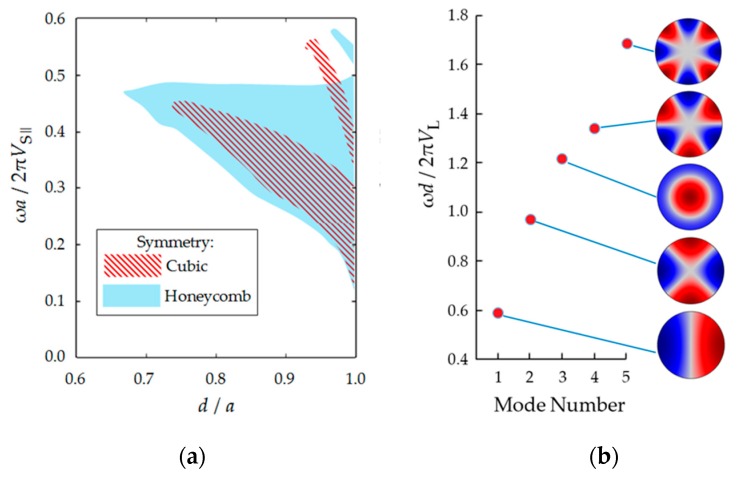
(**a**) Dependence of position and width of the full bandgap on the diameter of the scatters *d* and the lattice constant *a* in stainless steel matrix with cubic and honeycomb symmetries; (**b**) eigenmodes of liquid-filled cylinders.

Figure 2a shows the computed diagram showing the dependence of the full bandgap of a phononic crystal on the ratio of hole diameter and lattice constant. 

The plots are made for the cases of cubic and honeycomb symmetries of a phononic crystals. The frequency scale is normalized by multiplying by the distance between the holes and dividing by the longitudinal speed of sound in steel ($V_{S∥}$). $V_{S∥}$ is associated with the mechanical properties of a solid, Equation (6):

(6) $$V_{S∥}=\sqrt{\frac{E\left(1-\upsilon \right)}{\left(1+\upsilon \right)\left(1-2\upsilon \right)\rho_{S}}}.$$

where *E* is the Young’s modulus; υ is Poisson’s ratio. 

Figure 2b shows the resonant modes in the liquid cylinders. These modes were computed for the case of a resonator with perfectly reflective walls (free reflective boundary conditions). The frequencies are normalized by multiplying by the diameter of the hole and dividing by the speed of sound in the liquid. 

The frequency of resonant modes in cylindrical liquid resonators and the position of the band gaps on the frequency scale are matched by appropriate geometric parameters of the structure.

### 2.2. Experimental Setup

**Figure 3:**
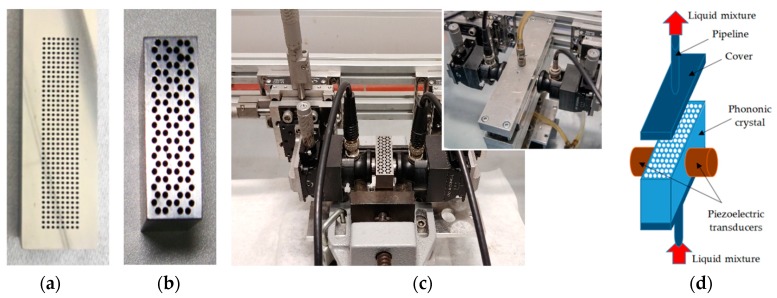
The steel plate with cubic (**a**) and honeycomb (**b**) periodic arrangement of cylindrical holes; (**c**) experimental setup; (**d**) schematic representation of the experimental setup.

The sensor consists of a phononic structure, clamp-on contact piezoelectric transducers (Panametrics V103-RB, central frequency 1.0 MHz) and a fluidic system (Figure 3c,d). The steel matrix can be partially or completely filled in with a liquid, depending on the approach described above. An Agilent 4395A network analyzer together with an Agilent 87511A S-parameter extension set was used to measure the S_21_-parameter of the structure. Figure 3a,b depicts the manufactured phononic crystals.

The diameter of the holes was much smaller than the height of the steel blocks to approach a 2D phononic crystal arrangement. Phononic crystal layouts, as well as parameters and dimensions were determined via numerical calculations. The robust design of the sensor meets the requirements for prospective industrial applications. In addition, for practical reasons, the size of the structure corresponds to frequencies in the range of 0.4–1 MHz. 

Mixtures of gasoline and ethanol, as well as water and propanol, were selected as sample liquids. The latter were used for calibration of the sensor. The material properties of these binary mixtures are well-studied and can be used as a reference for further investigations of more complex liquids [54]. The density of the composition varies monotonically, and the speed of sound is significantly nonlinear. Samples were prepared with a concentration of 1-propanol equal to 0, 5, 15, 25, 35, and 45% by volume.

A second part of experimental investigations focused on the application study made with 63–80 gasoline and 99.5% ethanol blends that were provided by Carl Roth GmbH and Sigma-Aldrich Chemie GmbH. Samples were prepared with ethanol concentrations of 0, 2.5, 5, 7.5, and 10 percent by volume. Interest in the study of these mixtures has been provoked by the proliferation of new fuel brands (E5, E10 gasoline and others) since ethanol additives are nowadays widespread due to the desire to reduce emissions of products of incomplete combustion of fuel in an internal combustion engine, as well as the search for an alternative way to increase the octave number and, accordingly, lower production costs.

## 3. Results

### 3.1. Narrowband Structure Design

Potentially, both types of sensor structures can be designed using both cubic and honeycomb symmetries of a phononic crystal. Since the width of the stopband in the honeycomb structure is much larger than in the cubic one with the equal filling factors (Figure 2a), it is more convenient to realize the first type of sensor based on honeycomb symmetry. By contrast, the cubic structure has an appropriate separated passband region that is featured with a narrow bandgap. Therefore, this design is more convenient to create a sensor of the second type with cubic symmetry.

#### 3.1.1. Narrow Transmission Band Structure

**Figure 4:**
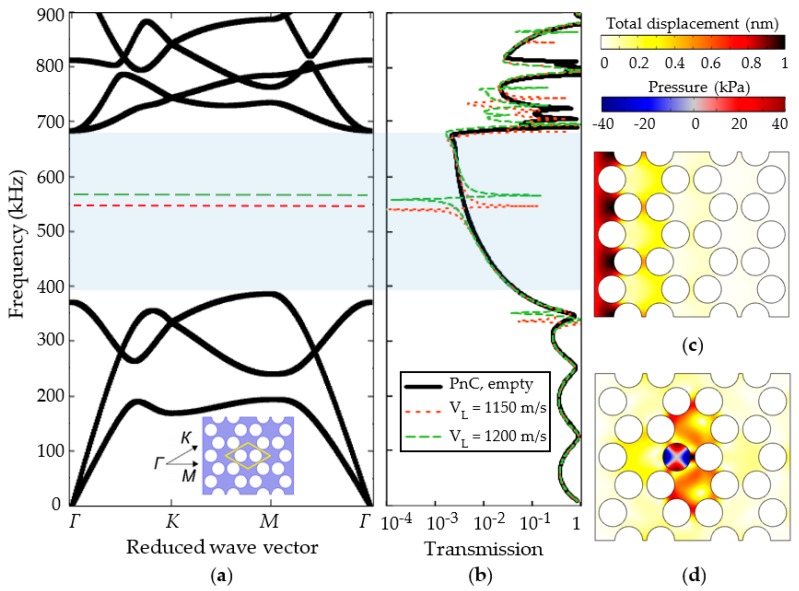
Theoretical results of the first type of phononic crystal sensor structure: (**a**) Band diagram for a 2D honeycomb infinite phononic crystal made of stainless steel with periodic hollow cylinders in a honeycomb arrangement (black solid curves) and localized substitution type defect state inside of the bandgap associated with liquid-filled hole for two different speed of sound values (green and red dotted curves); (**b**) transmission spectrum of the regular finite phononic crystal structure (black solid curve) and the structure with a liquid-filled defect for the two different speeds of sound; (**c**) total displacement distribution of the honeycomb-type phononic crystal structure exited at the boundary in the direction of wave propagation at the mid-bandgap frequency; (**d**) total displacement distribution of the phononic crystal structure with the liquid-filled local defect at the frequency of the second spinning pressure mode. The minimum of displacement for both demonstrated cases correspond to the white color.

The computation results of the band diagram and the transmission spectrum of a narrowband phononic structure of the first type are shown in Figure 4. Figure 4a shows a band diagram for a two-dimensional infinite structure of a honeycomb symmetry. The phononic crystal is made of stainless steel with a periodic arrangement of hollow cylinders (solid bold curves). The cylinder diameter is 2 mm and the lattice constant is 2.3 mm. The periodic structure has a wide bandgap in the frequency range from 400 to 690 kHz. When realizing a local defect by filling one of the holes with a liquid, an isolated localized state in the bandgap is created. For typical values of the gasoline speed of sound (about 1200 m/s [55,56,57]), mode number 2 lies in the center of the bandgap. This is the second spinning mode [58]. This defect mode is shown in the band diagram by a dotted line. Due to the broad bandgap structure, the pressure resonant mode can be found in the bandgap for a broad variety of speeds of sound of liquids, which is a valuable feature in terms of sensing. 

Figure 4b shows the transmission spectrum of a finite regular structure of a phononic crystal (solid line) and a finite structure with one scatter that is locally filled in with a liquid for two cases of sound velocities (dashed curves). The finite structure constitutes eight periods of hollow cylinders. The band diagram of the infinite crystal and the transmission spectrum of the finite structure are brought together in Figure 4b. It shows that the regular finite structure has a number of transmission peaks in the entire frequency range. The transmission of the finite structure is at least two orders of magnitude lower in the frequency range of the bandgap of the infinite phononic crystal. Figure 4c presents the displacement field distribution taken at the bandgap frequency. It shows that eight periods of the structure feature already a sufficient bandgap. When the central inclusion is locally filled with a liquid, the finite structure has an isolated narrow transmission peak (Figure 4b), which corresponds to the distribution of the displacement field shown in Figure 4e. A cylindrical liquid-filled resonator is surrounded by a periodic structure that provides high acoustic contrast. As a result, a high Q resonance can be achieved. In this case, the resonance peak turns out to be isolated, since there are no other modes to couple. When the composition of the fluid changes (more precisely, the speed of sound of the composition), the resonant frequency of the defect mode follows the variation of the speed of sound.

**Figure 5:**
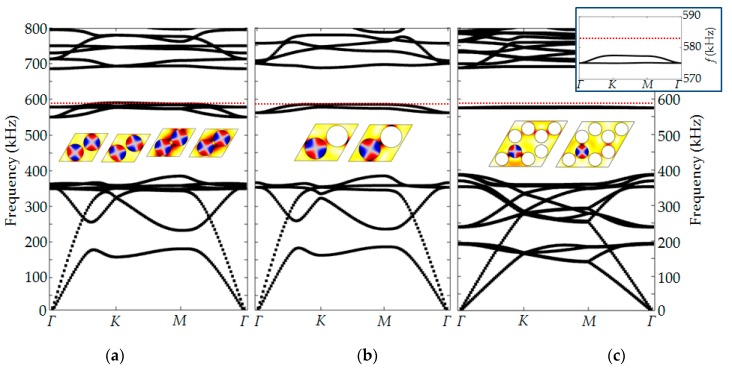
Band diagrams of honeycomb type phononic structure with liquid inclusions: (**a**) All the structure scatters are filled by a liquid; (**b**) every second scatter of a translation cell is liquid-filled; (**c**) a supercell with one liquid inclusion. The inset figure is a zoomed view on a supercell narrowband. The red dotted line shows the second resonant mode of a cylindrical liquid-filled resonator with perfect reflective boundaries. The distribution of displacement and pressure fields within unit cells for the defect eigenmodes are shown for the *Γ* point (*k* = 0).

The number of periods of designed finite structures is determined by two factors. On the one hand, it has to be sufficient to substantially attenuate the wave propagation through the structure. On the other hand, the transmittance must be greater than zero in order to be able to excite and detect the defect modes. We have studied several cases. Figure 5a shows a band diagram of a honeycomb phononic crystal with all scatters filled in with liquid. In this case, the structure bandgap disrupts with several coupled eigenmodes that assemble into a narrow passband. Reduction of the number of liquid inclusions lowers structure eigenmodes, but still supporting the existence of a passband in a middle of the bandgap. Figure 5c show the results for the supercell of 7/8 empty and 1/8 liquid-filled scatters. In this case the passband narrows significantly, however, it is still a narrow passband rather than a localized liquid pressure resonance. In an enlarged inset, one can see that the narrowband states have a wave vector dispersion that is not expected for a local liquid pressure resonance of cylindrical inclusion surrounded by a bandgap structure. On the displacement pictures the coupling modes are still recognizable, but less pronounced. At the same time, we can see the line that corresponds to pressure resonance of cylindrical volume, which is close to the edge of the narrowband obtained for a supercell. This concept with a locally liquid-filled inclusion is the most appropriate solution in terms of sensing applications and was experimentally verified in this work.

#### 3.1.2. Narrow Bandgap Structure

**Figure 6:**
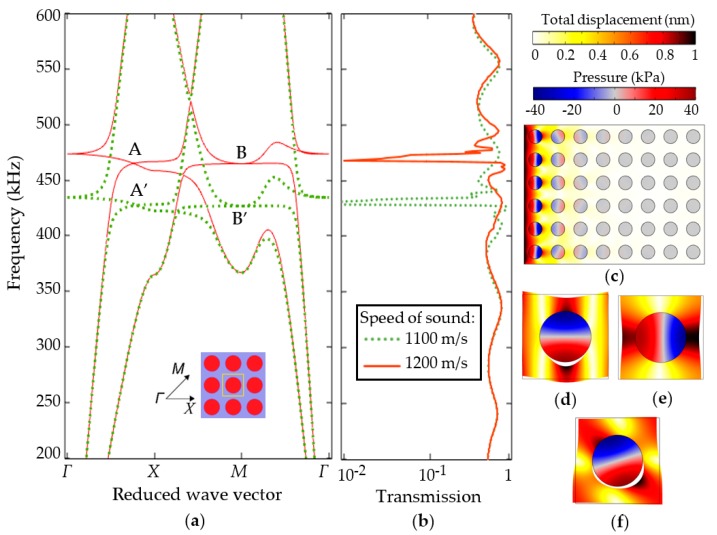
Theoretical description of the second type solid-liquid periodic composite sensor structure: (**a**) Band diagram for the 2D cubic infinite structure made of steel with periodic cylindrical liquid-filled holes computed for two different sound velocities in liquid (green dotted and red solid curves); (**b**) transmission spectrum of finite 2D solid-liquid composite structure with cubic symmetry for the same sound velocities; (**c**) displacement/pressure of the structure excited in the direction of wave propagation at the frequency corresponding to the transmission dip; (**d,e**) structure eigenmodes of solid-liquid composite unit cell corresponding to point A; (**f**) eigenmode of solid-liquid unit cell corresponding to point B. Total displacement and pressure distributions are shown by colors with white corresponding to the nodal lines or zero displacement.

Figure 6 demonstrates the computational results of the second type sensor structure. Figure 6a shows the band diagram of a cubic symmetry periodic structure with a cylindrical scatters filled with a liquid. A transmission spectrum of a finite structure with eight periods is shown in Figure 6b. Both, band diagram and transmission spectrum were again computed for two different velocities of sound of the constituting liquid. The diameter of the scatters is 1.5 mm, the lattice constant is 2.5 mm. Bringing together the band diagram and a transmission spectrum, we can see that the structure exhibits a high reflection in a narrow frequency region that corresponds to a low density of states. The A and A’ points in the band diagram correspond to the degenerative structure modes (Figure 6d,e). An interesting point is that the nodal line shown in Figure 6d is centered and in Figure 6e it is shifted relative to the center. This is due to the splitting of the vibration modes at points A and A’. In the system of non-interacting cells of liquid resonators, natural oscillations of Figure 6d,e would be equivalent, would have the same energy and, accordingly, frequency. Due to the interaction between the cells of the periodic liquid-solid composite structure, one finds a lifting of degeneracy. Point B and B’ is characterized by a hybrid mode when the acoustic wave propagates diagonally. Points A and B are the points of the minimum density of states of the structure. The range of the wave vector from point *Γ* to point *X*, which includes point A, is of greatest interest. It corresponds to the direction of propagation of the acoustic wave from the left to the right side of the periodic solid-liquid composite structure. Thus, the effect of lifting of the degeneracy opens a narrow stopband that can be found in a transmission spectrum as a transmission dip.

### 3.2. Experimental Results

**Figure 7:**
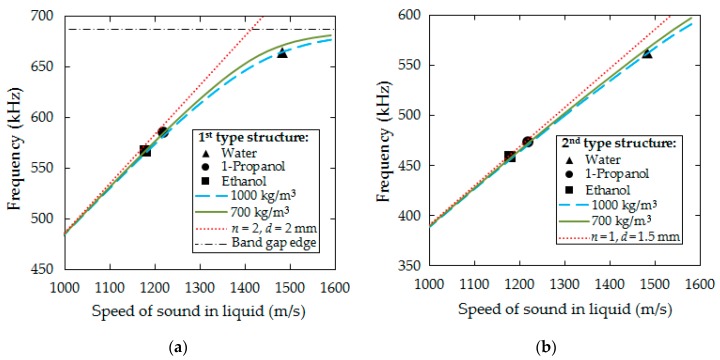
Dependence of the resonant frequency of the sensor on sound velocity of the liquid for two values of liquid density: (**a**) First type sensor; (**b**) second type sensor. The curves show the results of theoretical calculations, the markers show the results of experimental measurements for water, 1-propanol and ethanol. The red dotted lines show the dependence for the *n*-resonant mode of a cylindrical liquid-filled resonator of diameter *d* with perfectly reflective walls.

Binary mixtures of water-propanol, as well as gasoline and ethanol blends were selected for experimental studies. Figure 7 compares the theoretical dependence of the transmission peak position on the frequency scale on speed of sound of the constituting liquid for the sensor structures of first and second type, respectively, and experimental values for water, 1-propanol, and ethanol. The curves in the low-frequency region can be linearly approximated and are close to the curve of the isolated liquid resonator. The nonlinearity appearing closer to the edge of the bandgap is the consequence of coupling with structure eigenmodes. The influence of liquid density is rather weak.

**Figure 8:**
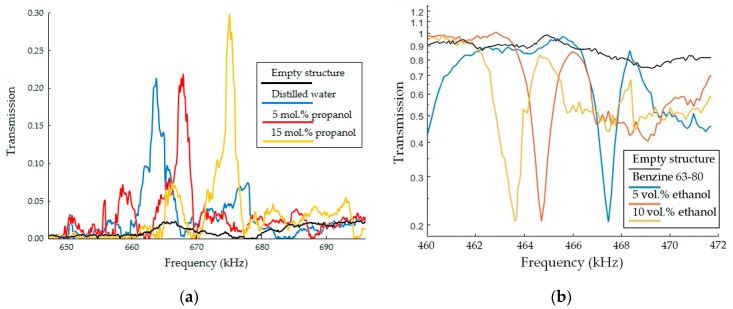
Experimental transmission frequency spectra of the two different sensors: (**a**) The first type of structure—the honeycomb phononic crystal with substitution type defect in form of a cylindrical hole filled with water-propanol mixtures for concentrations of 1-propanol 0, 5, and 15% by volume; (**b**) the second type of structure—solid-liquid cubic periodic arrangement of cylindrical holes filled with gasoline-ethanol mixtures for concentrations of ethanol 0, 5 and 10% by volume.

Figure 8 shows the transmission spectra measured for both structures. The structure of the first type produces isolated transmission peaks, while the structure of the second type transmission dips. Their positions are in accordance with the theoretical predictions.

We used well-studied water-propanol mixtures [54] for calibration and verification of the sensor, gasoline-ethanol mixture for determining the potential use of the phononic crystal liquid sensor.

**Figure 9:**
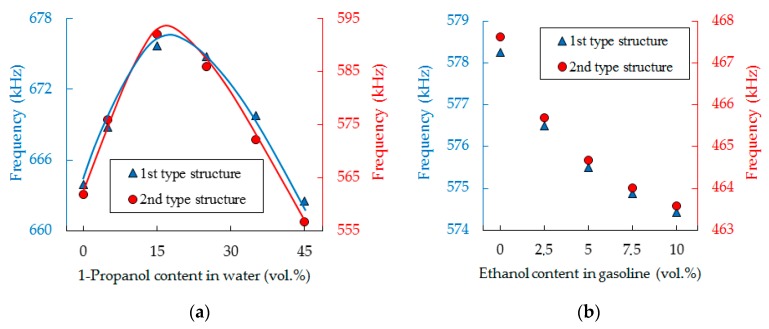
Summary plots for the two different experimental sensor responses on water-1-propanol (**a**) and gasoline-ethanol (**b**) mixtures. The solid lines show the modeling result based on data published in [54]. Markers show our experimental data.

A comparison of theory and experiment is demonstrated in Figure 9a, where the solid curves show the theoretical relation between resonant frequency of sensor and the content of propanol in water, the markers represent experimental data. The graphs obtained theoretically and the experimental results are in a good agreement. It should be noted, that the dependence of the resonant frequency on the speed of sound in liquids has a monotonic behavior (Figure 7), whereas the dependence of the speed of sound on the composition of the mixture can be significantly nonlinear, as demonstrated in Figure 9. Nonlinear mixing of water and propanol is due to a change in molecular structure. When 1-propanol is added to water, its properties are affected in two ways [59]: first, the 1-propanol acts as one of the components and thus imparts its physical and chemical characteristics; and second, the 1-propanol modifies the molecular structure of water. Since 1-propanol has a lower speed of sound than water, its addition to water should reduce the speed of sound. This is true only when the 1-propanol is present in large concentrations in the solution. In the water-rich region, the mixture shows anomalous behavior, which can be explained qualitatively by taking into consideration the solute-solvent interaction between 1-propanol and water [59]. The nonlinear effect for ethanol-gasoline binary mixtures is a result of interactions of hydrogen atoms in the homologues group C*_m_*H_2*m*+2_, which prevail in the gasoline we use, and hydroxyl (R-OH), which polarizes the alcohol molecule. Ethanol mixtures with non-polar solvents have non-linear isentropic compressibility and excess molar volume dependences from ethanol concentration with considerably rapid changes in a range of low ethanol concentrations [28,60]. Isentropic compressibility and the speed of sound are correlated, i.e., nonlinear behavior of thermodynamic characteristics and excess properties of ethanol–hydrocarbon binary mixtures are reflected in the speed of sound of the mixtures thereof. The sound velocity of gasoline measured in the phononic structures is about 1204 m/s at a temperature of 20 °C, which agrees well with the literature data [55,56,57].

## 4. Discussion

In this study the two cases are shown: a phononic crystal with a narrow transmission band inside a wide bandgap, and a narrow bandgap in a wide transmission band. Important criteria are a high sensitivity of the sensor response to the sound velocity of a liquid, a high quality factor of the resonance peak or dip, and the separation of it within the measured frequency band. Measurements of mixtures of water and 1-propanol, as well as gasoline and ethanol, have shown that the sensor has a sufficient sensitivity to measure the speed of sound of liquid mixtures.

The frequency response of both cases studied features narrow bands that respond to the variation of sound velocity of the liquid analytes. The transduction scheme of the acoustic part is linear except at the edges (Figure 7) because of the interaction of the resonances in the liquid-filled holes with solid-state eigenmodes. In case of the first type of sensor, the defect mode interacts with the edge of the passband, which creates a system of coupled oscillators with the corresponding frequency dispersion. Therefore, for an ideal liquid resonator (with perfectly reflective walls) the eigenfrequency depends linearly on speed of sound, but not on density. In our real case, a weak density dependence appears due to the energy exchange between the liquid and the solid-state matrix. This effect further reduces the sensitivity to the speed of sound. In the case of the second type of sensor, the nonlinearity in the response is caused by a change in the density of states in the acoustic spectra that begins to increase with increasing frequency.

To avoid the above nonlinear effects of both sensor types, the following design rules can be applied. In the case of the first type of structure the working range of the sensor should be deep in the bandgap. This can be done by adjusting the dimensions of the defect inclusion (Figure 2b), or by manipulating the bandgap width by changing the filling factor (Figure 2a). In the case of the second type of structure the narrow resonance reflection band should be placed in the frequency range with a lower density of the vibrational modes of the solid-state matrix. When the above conditions are met, the isolation of the resonant peaks/dips is achieved. The dependence of the resonant frequency on the speed of sound tends to be close to that of the ideal liquid-filled cavity resonator (Figure 7).

The key idea is not to prevent interactions completely. Instead, some interaction of the resonator with the structure is required to be able to detect the resonance. Thus, we need a method to transfer a small part of the resonance energy to the receiving transducer. As we have already discussed above, the transfer of energy using solid-state eigenmodes is undesired. The transmission through the interacting liquid resonators remains more attractive. When a set of non-interacting liquid resonators with identical geometry have a degenerated frequency spectrum, then the degeneracy of liquid resonators with some interaction gets lost. The *f*(*k*) branches in the band diagram split and a band of nonzero width appears. By decreasing the ratio of liquid-filled and empty holes (see Figure 5), we decrease the width of the strip, but at the same time we weaken the interaction between the liquid resonators. The optimal ratio allows for a narrow bandwidth in the bandgap and maintains a weak interaction between adjacent liquid-filled holes or with (geometric) edges of a finite structure.

The opposite principle must be applied in the case of the second type sensor, where we also search for the minimum width of splitting. Since all holes are liquid-filled, the design parameter is the ratio of the hole diameter to the lattice period to realize a band diagram depicted in Figure 6.

The sensitivity (Δ*f*/Δ*V*_L_) achieved in experimental studies was 0.4 kHz/(ms^−1^), the quality factor was around 600 for the samples of 1 cm height and 2000 for 4 cm height. 

The quality factor of the resonance peaks/dips is reduced by liquid viscosity, imperfections of the manufactured device, as well as radiation losses of acoustic energy at the ends of the cylindrical holes. We found that it depends on the height of phononic crystal plates. Thus, the main losses are acoustic energy radiation at the ends of the cylinders not considered in the 2D case. 

## 5. Conclusions

In this work we have introduced narrow band solid-liquid phononic crystal arrangements for liquid sensing purposes. We demonstrate two different concepts to design narrow band structures, one having a narrow passband, the other having a narrow stopband. We have shown the results of experimental investigations that confirm theoretical predictions.

In contrast to most previous studies, we show that liquid-induced eigenmodes of narrow bands do have a wave vector dispersion. Therefore, they cannot be considered as only a liquid pressure resonance. Liquid-induced structural resonances assemble the narrowband feature. The first concept of locally liquid-filled sub-structures is close to defect-type structures. A narrow bandgap of degenerative states is a new concept that, to the best of our knowledge, has not yet been reported. The results of the work extend the field of phononic crystal liquid sensors that have a high potential for evaluation of liquid analytes.

## References

[1] Kushwaha M.S., Halevi P., Dobrzynski L., Djafari-Rouhani B. (1993). Acoustic band structure of periodic elastic composites. Phys. Rev. Lett..

[2] Sigalas M., Economou E.N. (1993). Band structure of elastic waves in two dimensional systems. Solid State Commun..

[3] Lu M.-H., Feng L., Chen Y.-F. (2009). Phononic crystals and acoustic metamaterials. Mater. Today.

[4] Deymier P.A. (2013). Acoustic Metamaterials and Phononic Crystals.

[5] Laude V. (2015). Phononic Crystals: Artificial Crystals for Sonic, Acoustic, and Elastic Waves.

[6] Khelif A., Adibi A. (2016). Phononic Crystals.

[7] Vasseur J.O., Deymier P.A., Chenni B., Djafari-Rouhani B., Dobrzynski L., Prevost D. (2001). Experimental and Theoretical Evidence for the Existence of Absolute Acoustic Band Gaps in Two-Dimensional Solid Phononic Crystals. Phys. Rev. Lett..

[8] Khelif A., Choujaa A., Djafari-Rouhani B., Wilm M., Ballandras S., Laude V. (2003). Trapping and guiding of acoustic waves by defect modes in a full-band-gap ultrasonic crystal. Phys. Rev. B.

[9] Khelif A., Choujaa A., Benchabane S., Djafari-Rouhani B., Laude V. (2004). Guiding and bending of acoustic waves in highly confined phononic crystal waveguides. Appl. Phys. Lett..

[10] Khelif A., Aoubiza B., Mohammadi S., Adibi A., Laude V. (2006). Complete band gaps in two-dimensional phononic crystal slabs. Phys. Rev. E.

[11] Benchabane S., Khelif A., Rauch J.-Y., Robert L., Laude V. (2006). Evidence for complete surface wave band gap in a piezoelectric phononic crystal. Phys. Rev. E.

[12] Wu T.-T., Huang Z.-G., Tsai T.-C., Wu T.-C. (2008). Evidence of complete band gap and resonances in a plate with periodic stubbed surface. Appl. Phys. Lett..

[13] Khelif A., Djafari-Rouhani B., Vasseur J.O., Deymier P.A. (2003). Transmission and dispersion relations of perfect and defect-containing waveguide structures in phononic band gap materials. Phys. Rev. B.

[14] Khelif A., Wilm M., Laude V., Ballandras S., Djafari-Rouhani B. (2004). Guided elastic waves along a rod defect of a two-dimensional phononic crystal. Phys. Rev. E.

[15] Vasseur J.O., Deymier P.A., Djafari-Rouhani B., Pennec Y., Hladky-Hennion A.-C. (2008). Absolute forbidden bands and waveguiding in two-dimensional phononic crystal plates. Phys. Rev. B.

[16] Khelif A., Mohammadi S., Eftekhar A.A., Adibi A., Aoubiza B. (2010). Acoustic confinement and waveguiding with a line-defect structure in phononic crystal slabs. J. Appl. Phys..

[17] Vasseur J.O., Deymier P.A., Frantziskonis G., Hong G., Djafari-Rouhani B., Dobrzynski L. (1998). Experimental evidence for the existence of absolute acoustic band gaps in two-dimensional periodic composite media. J. Phys. Condens. Matter.

[18] Khelif A., Deymier P.A., Djafari-Rouhani B., Vasseur J.O., Dobrzynski L. (2003). Two-dimensional phononic crystal with tunable narrow pass band: Application to a waveguide with selective frequency. J. Appl. Phys..

[19] Pennec Y., Djafari-Rouhani B., Vasseur J.O., Khelif A., Deymier P.A. (2004). Tunable filtering and demultiplexing in phononic crystals with hollow cylinders. Phys. Rev. E.

[20] Tanaka Y., Tomoyasu Y., Tamura S. (2000). Band structure of acoustic waves in phononic lattices: Two-dimensional composites with large acoustic mismatch. Phys. Rev. B.

[21] Wu F., Liu Z., Liu Y. (2002). Acoustic band gaps in 2D liquid phononic crystals of rectangular structure. J. Phys. D Appl. Phys..

[22] Sukhovich A., Jing L., Page J.H. (2008). Negative refraction and focusing of ultrasound in two-dimensional phononic crystals. Phys. Rev. B.

[23] Oltulu O., Mamedov A.M., Ozbay E. (2017). Wave propagation and acoustic band gaps of two-dimensional liquid crystal/solid phononic crystals. Appl. Phys. A.

[24] Lucklum R., Li J. (2009). Phononic crystals for liquid sensor applications. Meas. Sci. Technol..

[25] Lucklum R., Li J., Zubtsov M. (2010). 1D and 2D phononic crystal sensors. Procedia Eng..

[26] Lucklum R., Ke M., Zubtsov M. (2012). Two-dimensional phononic crystal sensor based on a cavity mode. Sens. Actuators B.

[27] Oseev A., Lucklum R., Ke M., Zubtsov M., Grundmann R. Phononic Crystal Sensor for Liquid Property Determination. Proceedings of the International Society for Optics and Photonics.

[28] Oseev A., Zubtsov M., Lucklum R. (2013). Gasoline properties determination with phononic crystal cavity sensor. Sens. Actuators B Chem..

[29] Villa-Arango S., Torres Villa R., Kyriacou A., Lucklum R. (2016). Cavity Resonance Sensor with Disposable Analyte Container for Point of Care Testing. IEEE Sens. J..

[30] Villa-Arango S., Betancur Sánchez D., Torres R., Kyriacou P., Lucklum R. (2017). Differential Phononic Crystal Sensor: Towards a Temperature Compensation Mechanism for Field Applications Development. Sensors.

[31] Oseev A., Mukhin N., Lucklum R., Zubtsov M., Schmidt M.-P., Steinmann U., Fomin A., Kozyrev A., Hirsch S. (2018). Study of liquid resonances in solid-liquid composite periodic structures (phononic crystals)—Theoretical investigations and practical application for in-line analysis of conventional petroleum products. Sens. Actuators B Chem..

[32] Ke M., Zubtsov M., Lucklum R. (2011). Sub-wavelength phononic crystal liquid sensor. J. Appl. Phys..

[33] Lucklum R., Zubtsov M., Ke M. (2012). Liquid Sensor Utilizing a Regular Phononic Crystal with Normal Incidence of Sound. IEEE Trans. Ultrason. Ferroelectr. Freq. Control.

[34] Estrada H., García de Abajo F.J., Candelas P., Uris A., Belmar F., Meseguer F. (2009). Angle-Dependent Ultrasonic Transmission through Plates with Subwavelength Hole Arrays. Phys. Rev. Lett..

[35] Molerón M., Serra-Garcia M., Daraio C. (2014). Acoustic Fresnel lenses with extraordinary transmission. Appl. Phys. Lett..

[36] Gómez-Lozano V., Rubio C., Candelas P., Uris A., Belmar F. (2016). Experimental Ultrasound Transmission through Fluid-Solid and Air-Solid Phononic Plates. Materials.

[37] Lucklum R., Zubtsov M., Oseev A., Schmidt M.-P., Hirsch S., Hagemann F. (2013). Towards a SAW based phononic crystal sensor platform. Proceedings of the 2013 Joint European Frequency and Time Forum & International Frequency Control Symposium (EFTF/IFC).

[38] Oseev A., Schmidt M.-P., Lucklum R., Zubtsov M., Hirsch S. (2015). Phononic crystal based liquid sensor governed by localized defect resonances. Proceedings of the 2015 IEEE International Ultrasonics Symposium (IUS).

[39] Lucklum R., Zubtsov M., Oseev A., Schmidt M.-P., Hirsch S. (2016). SAW Based Sandwich Phononic Crystal Sensor. Procedia Eng..

[40] Oseev A., Lucklum R., Zubtsov M., Schmidt M.-P., Mukhin N., Hirsch S. (2017). SAW-Based Phononic Crystal Microfluidic Sensor—Microscale Realization of Velocimetry Approaches for Integrated Analytical Platform Applications. Sensors.

[41] Schmidt M.-P., Oseev A., Lucklum R., Zubtsov M., Hirsch S. (2016). SAW based phononic crystal sensor, technological challenges and solutions. Microsyst. Technol..

[42] Lucklum R., Zubtsov M., Oseev A. (2013). Phoxonic crystals—A new platform for chemical and biochemical sensors. Anal. Bioanal. Chem..

[43] Amoudache S., Pennec Y., Djafari Rouhani B., Khater A., Lucklum R., Tigrine R. (2014). Simultaneous sensing of light and sound velocities of fluids in a two-dimensional phoXonic crystal with defects. J. Appl. Phys..

[44] Lucklum R., Zubtsov M., Pennec Y. (2015). Tubular Bell—New Class of (Bio)Chemical Microsensors. Procedia Eng..

[45] Lucklum R., Zubtsov M., Pennec Y., Arango S.V. (2016). Disposable phononic crystal liquid sensor. Proceedings of the 2016 IEEE International Ultrasonics Symposium (IUS).

[46] Mukhin N., Lucklum R. (2019). QCM based sensor for detecting volumetric properties of liquids. Curr. Appl. Phys..

[47] Flory P.J. (1965). Statistical Thermodynamics of Liquid Mixtures. J. Am. Chem. Soc..

[48] Nayeem S.M., Nyamathulla S., Khan I., Rao D.K. (2016). Investigation of molecular interactions in binary mixture (benzyl benzoate + ethyl acetate) at T = (308.15, 313.15, and 318.15) K: An insight from ultrasonic speed of sound and density. J. Mol. Liq..

[49] Altas M.C., Kudryashov E., Buckin V. (2016). Ultrasonic Monitoring of Enzyme Catalysis; Enzyme Activity in Formulations for Lactose-Intolerant Infants. Anal. Chem..

[50] Buckin V., Altas M.C. (2017). Ultrasonic Monitoring of Biocatalysis in Solutions and Complex Dispersions. Catalysts.

[51] Hickey S., Lawrence M.J., Hagan S.A., Buckin V. (2006). Analysis of the phase diagram and microstructural transitions in phospholipid microemulsion systems using high-resolution ultrasonic spectroscopy. Langmuir.

[52] Acheson D.J. (1991). Elementary Fluid Dynamics. J. Acoust. Soc. Am..

[53] Landau L.D., Lifshitz E.M. (2014). Fluid Mechanics. Landau and Lifshitz: Course of Theoretical Physics.

[54] Schaafs W. (1967). Molecular Acoustics. New Series Group II Landolt-Börnstein.

[55] Berryman J.G. (1993). Analysis of ultrasonic velocities in hydrocarbon mixtures. J. Acoust. Soc. Am..

[56] Wang Z., Nur A. (1991). Ultrasonic velocities in pure hydrocarbons and mixtures. J. Acoust. Soc. Am..

[57] Wegge R., Richter M., Span R. (2015). Speed of Sound Measurements in Ethanol and Benzene over the Temperature Range from (253.2 to 353.2) K at Pressures up to 30 MPa. J. Chem. Eng. Data.

[58] Rona A. (2007). The Acoustic Resonance of Rectangular and Cylindrical Cavities. J. Algorithms Comput. Technol..

[59] Sehgal C.M., Porter B.R., Greenleaf J.F. (1986). Ultrasonic nonlinear parameters and sound speed of alcohol-water mixtures. J. Acoust. Soc. Am..

[60] Papaioannou D., Zlakas D., Panaylotou C. (1991). Volumetric properties of binarymixtures. 1. 2-Propanone 4-2,2,4-trimethylpentane and n-heptane + ethanol mixtures. J. Chem. Eng. Data.

